# Supplementary material for: The Inhibitory Effects of Plant Derivate Polyphenols on the Main Protease of SARS Coronavirus 2 and Their Structure–Activity Relationship
Source: Molecules. 2021 Mar 30;26(7):1924. doi: 10.3390/molecules26071924 (PMC8036510; doi:10.3390/molecules26071924)
Supplement: Supplementary file 1 [file molecules-26-01924-s001.pdf]

Supplementary Materials

The Inhibitory Effects of Plant Derivate Polyphenols on the Main Protease of SARS Coronavirus 2 and Their Structure–Activity Relationship

Thi Thanh Hanh Nguyen [1], Jong-Hyun Jung [2], Min-Kyu Kim [2], Sangyong Lim [2], Jae-Myoung Choi [3], Byoungsang Chung [3], Do-Won Kim [4] and Doman Kim [1,5,*]

1 Institutes of Green Bioscience and Technology, Seoul National University, Pyeongchang-gun, Gangwon-do 25354, Korea; hara2910@snu.ac.kr

2 Radiation Research Division, Korea Atomic Engery Research Institute, Jeongeup 56212, Korea; Jungjh83@kaeri.re.kr (J.-H.J.); mkkim@kaeri.re.kr (M.-K.K.); saylim@kaeri.re.kr (S.L.)

3 Ottogi Sesame Mills Co., Ltd., Chungcheongbuk-do 27623, Korea; mjchoi@ottogism.co.kr (J.-M.C.); bschung@ottogism.co.kr (B.C.)

4 Department of Physics, Gangneung-Wonju National University, Gangneung 25457, Korea; Do.Won.Kim@cern.ch

5 Graduate School of International Agricultural Technology, Seoul National University, Pyongchang-gun, Gangwon-do 25354, Korea

* Correspondence: kimdm@snu.ac.kr; Tel.: +82-33-339-5720; Fax: +82-33-339-5716

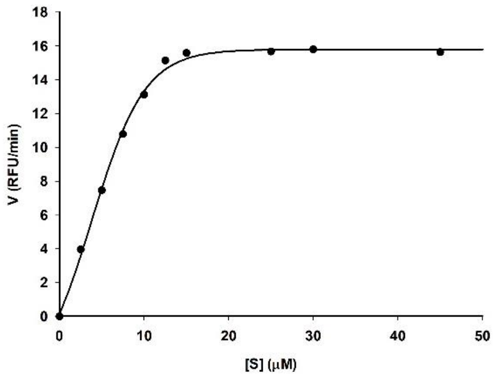

Figure S1 Michaelis-Menten plot for determination of K_m value of purified SARS-CoV-2 M^{Pro}.
